# GPT-4 as a Clinical Decision Support Tool in Ischemic Stroke Management: Evaluation Study

**DOI:** 10.2196/60391

**Published:** 2025-03-07

**Authors:** Amit Haim Shmilovitch, Mark Katson, Michal Cohen-Shelly, Shlomi Peretz, Dvir Aran, Shahar Shelly

**Affiliations:** 1 Department of Neurology Rambam Medical Center Haifa Israel; 2 Sagol AI Hub ARC Innovation Center Chaim Sheba Medical Center Ramat Gan Israel; 3 Department of Neurology Shamir Medical Center Be`er Ya`akov Israel; 4 Sackler School of Medicine Tel Aviv University Tel Aviv Israel; 5 Faculty of Biology Technion-Israel Institute of Technology Haifa Israel; 6 The Taub Faculty of Computer Science Technion-Israel Institute of Technology Haifa Israel; 7 Rapaport Faculty of Medicine Technion – Israel Institute of Technology Haifa Israel

**Keywords:** GPT-4, ischemic stroke, clinical decision support, artificial intelligence, neurology

## Abstract

**Background:**

Cerebrovascular diseases are the second most common cause of death worldwide and one of the major causes of disability burden. Advancements in artificial intelligence have the potential to revolutionize health care delivery, particularly in critical decision-making scenarios such as ischemic stroke management.

**Objective:**

This study aims to evaluate the effectiveness of GPT-4 in providing clinical support for emergency department neurologists by comparing its recommendations with expert opinions and real-world outcomes in acute ischemic stroke management.

**Methods:**

A cohort of 100 patients with acute stroke symptoms was retrospectively reviewed. Data used for decision-making included patients’ history, clinical evaluation, imaging study results, and other relevant details. Each case was independently presented to GPT-4, which provided scaled recommendations (1-7) regarding the appropriateness of treatment, the use of tissue plasminogen activator, and the need for endovascular thrombectomy. Additionally, GPT-4 estimated the 90-day mortality probability for each patient and elucidated its reasoning for each recommendation. The recommendations were then compared with a stroke specialist’s opinion and actual treatment decisions.

**Results:**

In our cohort of 100 patients, treatment recommendations by GPT-4 showed strong agreement with expert opinion (area under the curve [AUC] 0.85, 95% CI 0.77-0.93) and real-world treatment decisions (AUC 0.80, 95% CI 0.69-0.91). GPT-4 showed near-perfect agreement with real-world decisions in recommending endovascular thrombectomy (AUC 0.94, 95% CI 0.89-0.98) and strong agreement for tissue plasminogen activator treatment (AUC 0.77, 95% CI 0.68-0.86). Notably, in some cases, GPT-4 recommended more aggressive treatment than human experts, with 11 instances where GPT-4 suggested tissue plasminogen activator use against expert opinion. For mortality prediction, GPT-4 accurately identified 10 (77%) out of 13 deaths within its top 25 high-risk predictions (AUC 0.89, 95% CI 0.8077-0.9739; hazard ratio 6.98, 95% CI 2.88-16.9; *P*<.001), outperforming supervised machine learning models such as PRACTICE (AUC 0.70; log-rank *P*=.02) and PREMISE (AUC 0.77; *P*=.07).

**Conclusions:**

This study demonstrates the potential of GPT-4 as a viable clinical decision-support tool in the management of acute stroke. Its ability to provide explainable recommendations without requiring structured data input aligns well with the routine workflows of treating physicians. However, the tendency toward more aggressive treatment recommendations highlights the importance of human oversight in clinical decision-making. Future studies should focus on prospective validations and exploring the safe integration of such artificial intelligence tools into clinical practice.

## Introduction

The advent of GPT-4 [1], launched by OpenAI in March 2023, marked a significant milestone in the evolution of artificial intelligence (AI) and its applications in various domains, including health care. GPT-4, a model under the umbrella of GPT, exemplifies the advancement in large language model (LLM) technology [2,3]. The foundational architecture of this technology involves training on extensive datasets, enabling the model to function as a “few-shot learner.” This capability allows GPT-4 to adapt to new domains and continuously refine its performance through ongoing learning [2,4-6].

In the realm of clinical medicine, the potential applications of LLMs like GPT-4 are particularly intriguing. These models offer promise as supportive tools for health care professionals, aiding in the efficient summarization of patient data, assisting in decision-making processes, and potentially improving the accuracy and speed of medical interventions [7,8]. Recent research has underscored the capabilities of GPT-4 in complex medical tasks [9]. Notably, the model has demonstrated proficiency in examinations akin to the United States Medical Licensing Examination, achieving scores that meet or nearly meet the passing thresholds [10]. Additionally, in assessments modeled after neurology board exam questions, GPT-4 has shown a high accuracy rate, improving with repeated attempts [9,11,12].

The management of acute ischemic stroke (AIS) presents a critical and time-sensitive challenge in clinical settings. The approach to diagnosing and treating AIS requires a synthesis of information including patient symptoms, physical and neurological examinations, medical history, and imaging results. Despite the availability of established guidelines by the American Heart Association/American Stroke Association for stroke management [13-16], the pivotal role of the treating physician’s judgment remains. Variability in clinical presentations and the urgent need for decision-making underscore the potential value of AI-assisted tools in this context. Moreover, predicting early mortality in AIS is essential for guiding treatment decisions, optimizing resource allocation in health care settings, facilitating effective communication with patients and their families, supporting research and clinical trials, and contributing to quality improvement initiatives. In accordance, several traditional machine learning models have been trained for this task in recent years [17-20].

Here, we leveraged patient data from the emergency department (ED) of a large referral hospital, focusing on individuals presenting with stroke symptoms, to evaluate the effectiveness of GPT-4 in delivering accurate clinical decisions for the treatment of AIS. We also assessed its proficiency in predicting 90-day mortality outcomes. The aim of this study was to quantify the extent to which an advanced language model like GPT-4 can augment the clinical decision-making process in AIS management. Specifically, we hypothesized that GPT-4 could provide accurate treatment recommendations and mortality predictions comparable to those of human experts, potentially contributing to improved patient outcomes in one of the most critical areas of emergency medicine.

## Methods

### Cohort Selection

This retrospective study comprised 100 consecutive cases from the ED of Rambam Healthcare Campus. All patients treated between January 2022 and April 2023 received a confirmed diagnosis of AIS. The inclusion criteria encompassed patients aged older than 18 years, a National Institutes of Health Stroke Scale (NIHSS) [21] score of 5 or higher (with the exception of patient 93 who received tissue plasminogen activator [tPA] offsite), and less than 5 hours from symptom onset to undergoing a noncontrast computed tomography (CT) of the brain. All included patients underwent noncontrast brain CT, CT angiography, and CT perfusion while in the ED. This cohort was specifically chosen for its alignment with American Heart Association guidelines for acute stroke management [13], making each patient a potential candidate for both tPA and endovascular thrombectomy (EVT) treatment. A total of 17 patients not meeting these criteria were categorized as “complex” cases, in which the clinical scenario warranted extra consideration of off-guideline treatment options, and there was a need to assess the individual patient’s unique characteristics, medical history, and condition. For every patient, comprehensive medical records from their ED arrival, including imaging results, were collected and translated from Hebrew to English. Exclusion criteria were patients with incomplete clinical data or where stroke was not the final diagnosis.

Clinical data for each patient included demographics, medical history, chief complaints, symptom onset time, physical and neurological examinations, NIHSS score, imaging results (including Alberta Stroke Program Early CT Score [22] when available), treatment received, and mortality data. An experienced stroke specialist, blinded to the outcomes, reviewed the cases and made treatment decisions among no treatment, tPA, EVT, or a combination of tPA and EVT. All data were deidentified, removing identifiers, names, and dates.

### Analysis Pipeline

The analysis used the OpenAI application programming interface “create chat completion” method with the model gpt-4-1106-preview. Default parameters were set (temperature=1; top_p=1; n=1), and submissions were made using the R (R Foundation for Statistical Computing) wrapper library *openai*. Full prompt and example are available in Multimedia Appendix 1.

To assess the reliability of GPT-4 responses, each case underwent 5 submissions, as well as an additional submission without the accompanying clinical presentation narrative. For every treatment decision, GPT-4 provided a narrative explanation. In 95% (475/500) of cases, GPT-4 returned responses in the requested structure, which were automatically scraped with R. Unstructured responses were manually entered. For estimations provided as a range, the average was used. If GPT-4 provided a number with a greater symbol (eg, >50), the number was recorded with an additional 5. In 0.8% (4/500) of cases, GPT-4 did not return numeric responses for treatment decisions, and in 8.6% (43/500) of responses, it did not provide a 90-day mortality estimate.

### Statistical Analysis

GPT-4’s responses were scaled from 1 to 7 for treatment decisions and from 0 to 100 for 90-day mortality estimations. Averages were calculated across the 5 repeats. All statistical analyses were conducted using R (version 4.3.2), using base R functions, *predictive receiver operating characteristic (ROC)* 1.18.5, and *survival* 3.5.7. ROC curves were smoothed. Agreement between treatment decisions was measured using a linear weighted Cohen κ coefficient, using the *psych* 2.3.12 library.

### Ethical Considerations

This study was approved by the Rambam Medical Center Helsinki Committee (0156-24-D) as a retrospective analysis. The requirement for informed consent was waived due to the retrospective nature of the study and the use of deidentified data. All patient information was anonymized prior to analysis, with all identifiers, names, and dates removed to ensure privacy and confidentiality. No compensation was provided to participants as this was a retrospective study using existing clinical data. The study did not involve any images that could potentially identify individual participants. This research was conducted in accordance with the principles of the Declaration of Helsinki and adhered to all relevant institutional and national research ethics guidelines.

## Results

### Patient Demographics and Clinical Data

**Figure 1:**
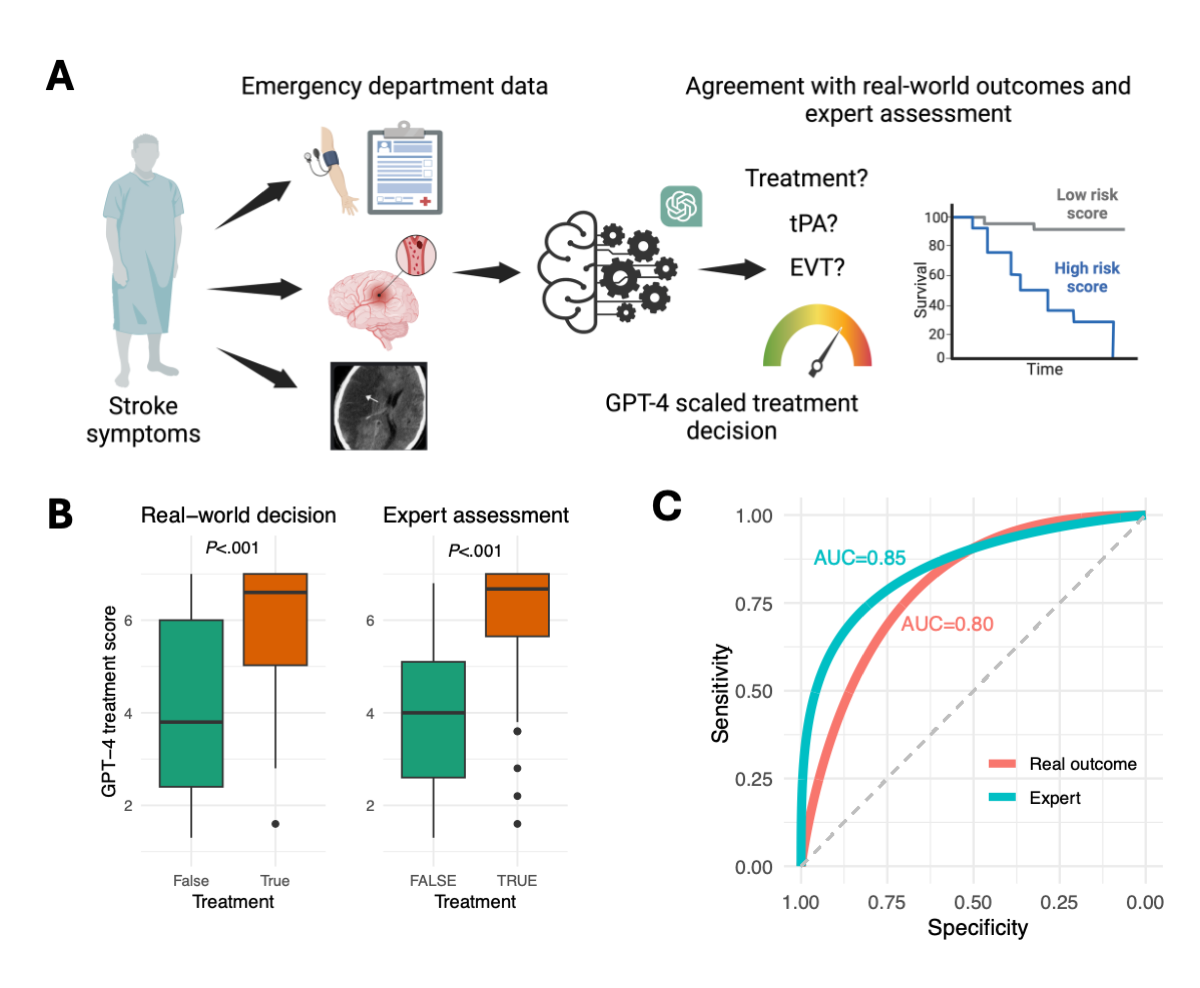
Study design and GPT-4 performance evaluation. (A) Illustration of the study design involving 100 consecutive patients with stroke who underwent a comprehensive stroke workup, including perfusion, angiography, and noncontrast brain CT upon arrival at the emergency department. Clinical information, demographics, comorbidities, and CT perfusion results were recorded. The textual reports from these investigations were entered into the GPT-4 API, which was instructed to provide scores indicating whether to treat the patient, whether to administer tPA, whether to pursue EVT, and an estimate of 90-day mortality. (B) Box plots presenting average scores of GPT-4 assessments for decision to treat (y-axis). The comparison is made against real-world decisions and expert assessments of each case (true: to treat the patient and false: to not treat). (C) ROC curves and AUC scores of GPT-4 average scores for decision to treat, compared to real-world decisions and expert assessments. API: application programming interface; AUC: area under the curve; CT: computed tomography; EVT: endovascular thrombectomy; ROC: receiver operating characteristic; tPA: tissue plasminogen activator.

We generated a cohort from 100 consecutive cases of patients presenting with acute stroke symptoms at the ED of Rambam Healthcare Campus. All cases underwent full clinical and radiological evaluation in the emergency setting for acute stroke and were fully evaluated by a neurologist (Table 1 and Figure 1A). Revascularization treatment was administered to 78 of the patients: 36 were treated with tPA, 30 with EVT, and 12 received both. Within this cohort, 13 patients died within 90 days and 21 in total. Overall, 17 cases were classified as “complex” when not fitting exact treatment guidelines [13]. The data for each case encompassed demographics, NIHSS [21] scores, the timing of arrival to brain CT, onset of symptoms, and details from textual brain imaging results and risk factors that were available as medical history at the time of admission to the ED (Table S1 in Multimedia Appendix 2).

**Table 1:** Study cohort clinical information and demographics.

Variable			Simple cases (n=83)		Complex cases (n=17)
Female sex, n (%)			38 (46)		7 (41)
Age (years), median (IQR)			75.0 (68.0-79.5)		71.0 (65.0-77.0)
First NIHSS^a^, median (IQR)			12.0 (8.5-16.5)		5.0 (5.0-9.0)
Time to CT^b^ (hours), median (IQR)			1.8 (I1.5-2.6)		4.45 (3.0-5.2)
**Brain CT findings, n (%)**					
	LVO^c^	48 (58)		7 (41)	
	MCA^d^	47 (57)		4 (24)	
	PCA^e^	8 (10)		4 (24)	
**Risk factors, n (%)**					
	Hypertension	51 (61)		10 (59)	
	DM^f^	35 (42)		3 (18)	
	Dyslipidemia	36 (43)		6 (35)	
	Smoking	11 (13)		4 (24)	
	CKD^g^	11 (13)		0 (0)	
	Obese	5 (6)		0 (0)	
	Cancer	9 (11)		1 (6)	
	HF^h^	7 (8)		1 (6)	
	Cardiac arrhythmia	19 (23)		2 (12)	
	Family history for CAD^i^	1 (1)		0 (0)	
tPA^j^, n (%)			29 (35)		7 (41)
EVT^k^, n (%)			29 (35)		1 (6)
tPA + EVT, n (%)			12 (14)		0 (0)
90-day mortality, n (%)			11 (13)		2 (12)
Overall mortality, n (%)			17 (20)		4 (24)

^a^NIHSS: National Institutes of Health Stroke Scale.

^b^CT: computed tomography.

^c^LVO: large vessel occlusion.

^d^MCA: middle cerebral artery.

^e^PCA: posterior cerebral artery.

^f^DM: diabetes mellitus.

^g^CKD: chronic kidney disease.

^h^HF: heart failure.

^i^CAD: coronary artery disease.

^j^tPA: tissue plasminogen activator.

^k^EVT: endovascular thrombectomy.

A stroke specialist, blinded to the outcomes, retrospectively reviewed each case. In 82 of the cases, the expert’s decisions aligned with the actual treatments administered. Of note, the expert recommended not treating 11 patients who received treatment and suggested treatment for 7 who did not receive any. Concerning specific treatments, full agreement was observed in 61 cases, although the expert more frequently recommended combining tPA and EVT than what was observed in practice (Cohen κ=0.51, signifying moderate agreement).

### GPT-4 Clinical Decisions

Independently, each case was assessed with GPT-4, generating a treatment recommendation scale from 1=intervention not recommended to 7=highly recommended (Figure 1A; Table S2 in Multimedia Appendix 2). To account for the variability in GPT-4 responses, each case was assessed 5 times. Cohen κ for treatment scores across runs ranged from 0.56 to 0.73. As expected, the predefined “complex” cases demonstrated significantly greater variance between runs (*P*=.02).

Comparing GPT-4’s treatment scale to both the expert’s decision and the actual treatment revealed that the average scores from GPT-4 for patients who were treated were, on average, 1.9 points higher than those not treated (*P*<.001), and there was a 2.1-point difference in comparison to the expert decision (*P*<.001; Figure 1B). The average scores provided an area under the ROC curve (AUC-ROC) of 0.80 (95% CI 0.69-0.91) compared to the real-world decision, and 0.85 (95% CI 0.77-0.93) compared to the expert decision (Figure 1C). These average scores for AUCs were higher than those of each independent run (Multimedia Appendix 3). Additionally, removing the clinical presentation narrative from GPT-4’s analysis resulted in a drop in AUC to 0.70 with the real-world decision and 0.72 with the expert decision (Multimedia Appendix 3), highlighting the importance of unstructured narrative data in treatment decision-making. Similarly, setting the temperature of GPT-4 to 0 resulted in AUCs of 0.70 and 0.72 with the real-world and expert decisions, respectively, suggesting the need to allow GPT-4 more creativity to obtain better decisions.

Using a score threshold of 4, we observed 22 disagreements between GPT-4 and the real-world treatment and 20 disagreements with the expert decision. Notably, a substantial proportion of these disagreements coincided with cases where the expert and real-world decisions diverged, with 18 (60%) out of 30 such cases showing this dual disagreement. Moreover, complex cases were more prone to discrepancies, as 7 disagreements with the real-world decision and 5 with the expert decision were noted among the 17 complex cases. The specialist examined the explanatory text produced by GPT-4 for all discrepancies between the model and their blinded assessments, evaluating whether they agreed that the explanatory text, as part of the original model output, was logical and could be deemed good practice. Of the 20 instances where disagreements occurred, in 3 cases, the expert, after having carefully considered GPT-4’s detailed explanations, conceded that GPT-4’s assessment was preferable to their original decision. In additional 2 cases, the expert acknowledged that GPT-4’s suggested approach was indeed acceptable and aligned with viable treatment options. In instances where the expert disagreed with GPT-4’s reasoning, the disagreements primarily revolved around 3 key issues. First, GPT-4 inaccurately associated abnormal angiographic findings with clinical presentations. An illustrative case is that of a patient with stenosis of the right-sided middle cerebral artery who was presented with right hemiparesis (case 94). Despite these 2 elements potentially being anatomically unrelated, GPT-4 linked them erroneously. The second notable issue pertained to ethical considerations, particularly in a case involving a patient with active laryngeal cancer and cognitive decline. According to guidelines, the patient was deemed eligible for treatment, but the expert’s decision was to not proceed with treatment as life expectancy was short and he was palliative (case 14). Third, discrepancies arose in deviations from guidelines, particularly in cases of distal thrombectomies. For instance, in the case of a patient with M2 obstruction (considered distal thrombus) aged 96 years, GPT-4 recommended against treatment, which is the established guidelines; however, the expert call was to proceed with thrombectomy due to a high NIHSS score and good results in such cases in the past from personal experience (case 54).

**Figure 2:**
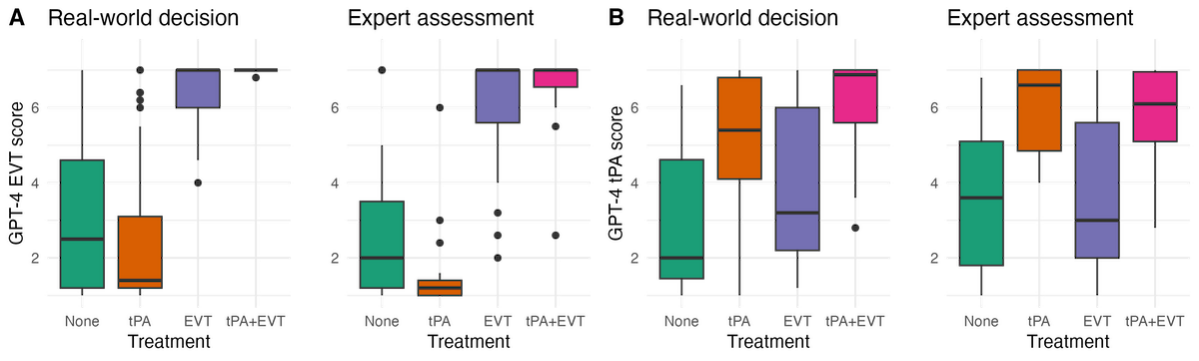
GPT-4 treatment type scores. Box plots depict GPT-4 treatment type scores, with the y-axis representing probability score (1-7 scale). Each treatment category is color coded: green for no intervention, orange for tPA, purple for EVT, and pink for tPA and EVT. (A) GPT-4 scores for EVT, stratified by real-world decisions and expert assessments. (B) GPT-4 scores for tPA, stratified by real-world decisions and expert assessments. EVT: endovascular thrombectomy; tPA: tissue plasminogen activator.

In assessing GPT-4’s ability to choose the best treatment option, it showed near-perfect agreement with real-world decisions in recommending EVT: GPT-4 suggested EVT for all patients (42/42, 100%) treated with EVT (average score>4). The expert suggested EVT for 55 patients, of which 50 were also recommended EVT by GPT-4, corresponding to an AUC of 0.94 (95% CI 0.89-0.98) with real-world decisions and 0.95 (95% CI: 0.90-0.99) with the expert (Figure 2A). For tPA treatment, GPT-4 recommended it for 38 (79%) of the 48 patients who received it, showing a closer agreement with the expert. Of the 41 patients recommended for tPA by the expert, GPT-4 agreed on 35 (85%), corresponding to an AUC of 0.77 (95% CI 0.68-0.86) with real-world decisions and 0.82 (95% CI 0.73-0.90) with the expert (Figure 2B).

### Mortality Risk

**Figure 3:**
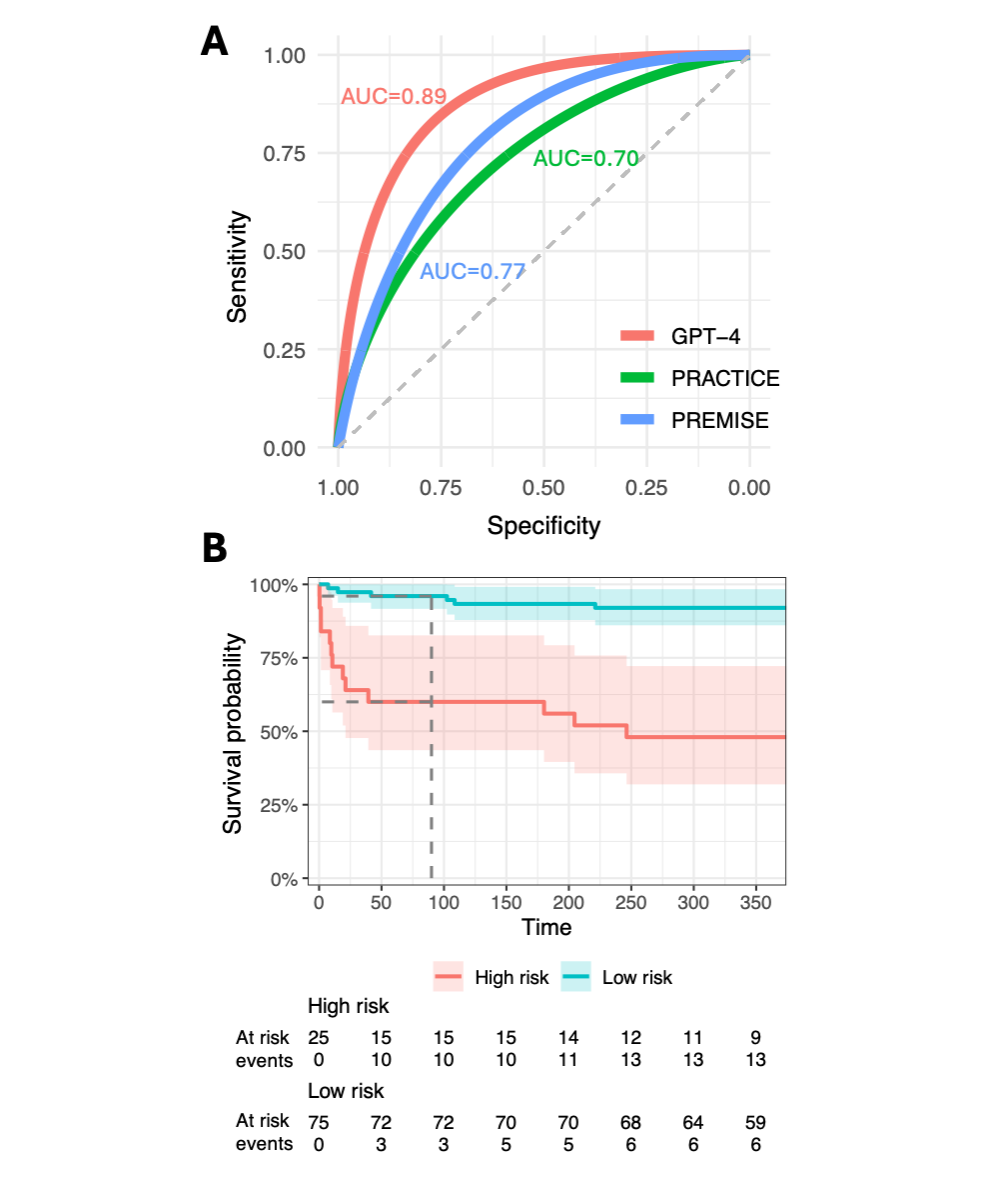
GPT-4 mortality predictions. (A) ROC curve for 90-day mortality estimations by GPT-4 (red), PRACTICE (green), and PREMISE (blue). (B) Kaplan-Meier plot stratifying individuals into low- and high-risk categories for mortality based on GPT-4’s 90-day mortality estimations. AUC: area under the curve; ROC: receiver operating characteristic.

We further evaluated the ability of GPT-4 to predict 90-day mortality. The model estimated an average mortality risk of 55.1% for patients who died within 90 days, compared to 31.5% for survivors (*P*<.001), yielding an AUC of 0.89 (95% CI 0.81-0.98; Figure 3A). To contextualize these results, we compared GPT-4’s performance with that of 2 recent machine learning models specifically trained for 90-day mortality prediction. In our cohort, the PRACTICE model [18] achieved an AUC of 0.70, significantly worse than the GPT-4 predictions (log-rank *P* value=.02), while the PREMISE model [19] reached an AUC of 0.77 (*P*=.07; Figure 3A). These comparisons underscore GPT-4’s remarkable accuracy in mortality risk assessment, outperforming specialized, trained predictive models.

For identifying high-risk patients, we set a threshold at the top 25% of the cohort, which corresponded to a predicted mortality risk cutoff of 41%. Within this high-risk group, 10 patients passed away within 90 days of admission, and an additional 3 within the subsequent year (Figure 3B). Conversely, among the remaining 75 patients categorized as lower risk, only 3 deaths occurred within the 90-day period, and 6 in total during the first year. The calculated hazard ratio was 6.98 (95% CI 2.88-16.9; *P*<.001), reinforcing the model’s capability to stratify patients based on their mortality risk effectively.

## Discussion

Here, we demonstrate the potential of GPT-4 as a clinical decision-support tool in AIS management. Our main findings show that treatment recommendations by GPT-4 closely aligned with both expert opinions (AUC 0.85) and real-world decisions (AUC 0.80). Notably, GPT-4 exhibited high accuracy in predicting 90-day mortality (AUC 0.89), outperforming specialized machine learning models.

AIS is a leading cause of mortality and disability worldwide [23-25]. The urgency of stroke care is particularly critical in regions with limited access to specialized stroke units or qualified physicians [26,27]. GPT-4’s ability to operate seamlessly within existing treatment routines, relying solely on routine chart information, makes it valuable for quick triage in underresourced settings [7]. This accessibility could democratize high-level medical consultation, extending expert-level decision-making to underresourced health care facilities.

In our study, GPT-4 demonstrated high accuracy in predicting 90-day mortality for patients with AIS undergoing endovascular treatment. The model used a diverse range of clinical and imaging variables, offering a more comprehensive approach compared to existing models like Houston intraarterial therapy, Houston intraarterial therapy 2, PREMISE, and PRACTICE [18,19,28,29]. Unlike traditional health care predictive models that rely on structured data, GPT-4 provided recommendations based on narrative text. Our analyses highlighted the significance of unstructured data, as evidenced by the drop in prediction accuracy when the narrative clinical presentation was excluded. This showcases GPT-4’s capability to handle complex medical data in a way that aligns with the natural flow of clinical information.

A crucial aspect of deploying AI models like GPT-4 in health care is the transparency and interpretability of their decision-making process. While GPT-4’s natural language outputs can give the impression of explainability, these may not necessarily reflect a truly reliable reasoning process. Our analysis focused on the face value of GPT-4’s rationales, which were deemed insightful by the expert reviewer. However, we acknowledge the potential for convincing but flawed explanations, a known limitation of LLMs. This highlights the importance of critical evaluation and cautious interpretation of such model outputs, particularly in high-stakes medical decision-making contexts. Ongoing research is needed to address the transparency and reliability of AI systems’ reasoning processes before their broader integration into clinical practice.

Despite its promising results, our study has several limitations. We must acknowledge certain challenges in applying GPT-4, especially regarding its ability to assess ethical issues. The model may face difficulties in addressing the nuanced and complex ethical considerations intrinsic to medical decision-making. This limitation emphasizes the necessity for cautious and supplementary human oversight when deploying AI tools like GPT-4 in sensitive health care contexts. The occurrence of “hallucinations” or erroneous outputs is another concern, although we demonstrated that running multiple assessments can mitigate this risk. Future research should focus on refining these methods to further reduce inaccuracies.

Another consideration is the generalizability of these findings. While it is possible that the recommendations may partially reflect the clinician’s intuition encoded in the clinical notes, our analyses suggest that the model’s assessments go beyond mere interpretation. The discrepancies observed between the GPT-4 recommendations and both the real-world treatment decisions and the expert evaluations indicate that the model is capable of making independent assessments based on the provided data. Furthermore, the clinical presentation notes and imaging report interpretations (Table S1 in Multimedia Appendix 2) do not explicitly convey the clinician’s treatment preferences or intuitions, suggesting that GPT-4 is not simply regurgitating the clinician’s thought process. Another possible limitation is the study’s exclusion criteria, particularly the retrospective exclusion of patients with incomplete clinical data or those who were ultimately diagnosed with conditions other than stroke. While these exclusions were necessary to ensure the study focused on accurately diagnosed AIS cases for which GPT-4 decision-support capabilities could be most relevant, we acknowledge that this approach may limit the generalizability of our findings to broader clinical settings. In real-world scenarios, clinicians are often faced with diagnostic uncertainty and incomplete information when making treatment decisions. Finally, our study was conducted in a single center with a specific patient population. Further studies across diverse settings and larger populations are necessary to validate the efficacy and applicability of GPT-4 in various clinical environments.

In conclusion, our study introduces a groundbreaking approach to clinical decision support in stroke management using GPT-4. This model has shown the potential to process narrative text, provide explainable recommendations, and enhance medical decision-making. As we continue to explore and refine this technology, it holds the promise of transforming patient care and improving outcomes in one of the most critical areas of medicine.

## Appendix

Multimedia Appendix 1 Prompt used.

Multimedia Appendix 2 Supplementary tables.

Multimedia Appendix 3 GPT-4 Assessments Performance. Area under the curve (AUC) for GPT-4 decision to treatment scores of each of the individual submissions (1-5) and the average. Each individual submission is lower than the average. In addition, we submitted the cases without the clinical presentation narrative, which yielded lower AUC (no narrative). Similarly, lower AUC was observed when cases were submitted with temperature=0.

## Abbreviations

AI: artificial intelligence
AIS: acute ischemic stroke
AUC: area under the curve
CT: computed tomography
ED: emergency department
EVT: endovascular thrombectomy
LLM: large language model
NIHSS: National Institutes of Health Stroke Scale
ROC: receiver operating characteristic
tPA: tissue plasminogen activator

## Data Availability

All data generated or analyzed during this study are included in Multimedia Appendix 2.
