# Supplementary material for: GPT-4 as a Clinical Decision Support Tool in Ischemic Stroke Management: Evaluation Study
Source: JMIR AI. 2025 Mar 7;4:e60391. doi: 10.2196/60391 (PMC11928773; doi:10.2196/60391)
Supplement: Multimedia Appendix 1 [file ai_v4i1e60391_app1.pdf]

Multimedia Appendix 1

The full prompt given to GPT-4 was as follows:

"Imagine you are a board-certified neurologist in the emergency room. You are receiving a clinical case. Describe the best neurological approach leading to the best neurological outcome, and the lowest chance of mortality. Base your decision on the current guidelines and reason your decision. Note that in some cases patients should not be treated ***although*** *the best treatment option due to the patient fragility.*

Here is your case: <<CASE>>

First, provide the full reasoning. Next, based on your response, answer the questions below. Only return a number, no additional reasoning. Provide the results in a structured format as following: [A, B,C,D], where A is answer for q1, B for q2, C for q3 and D for q4. For example, an answer could be [4,3,2,40].

1. Any intervention (tPA or EVT)? Answer with scale 1 to 7, where 1 is intervention not recommended and 7 is intervention is highly recommended.

2. Thrombolytic therapy (tissue plasminogen activator; tPA)? Answer with scale 1 to 7, where 1 is tPA not recommended and 7 is tPA is the best option.

3. Endovascular thrombectomy (EVT)? Answer with scale 1 to 7, where 1 is EVT not recommended and 7 is EVT is the best option.

4. What is your estimation for 90-day mortality probability? Provide estimation even if there is not enough information. Use the scale 0 and 100."

The <<CASE>> placeholder was replaced with relevant patient details in a structured "feature: value" format, using all the features available in Supplementary Table 1. Here is an example for Case #1:

"Here is your case: subject number: 100;Clinical Presentation: A 70-75 years old male with medical history of diabetes mellitus, IHD, COPD, past smoker. Regular medications include aspirin. Presented with left-sided weakness and slurred speech. Took aspirin before his arrival to the ER. .;Age: 70-75;sex: Male;NIHSS: 5 for mild facial

palsy, left-sided mild hemiparesis, mild hypoesthesia, mild dysarthria.;NIHSS: 5;Stroke 1=yes no=0: 1;Time from arrival to brain CT: 0.0469675925924093;Time of symptoms to NCCT (Hours): 0.21944444444444444;Time of symptoms to NCCT (Decimal hours): 5.26666666666667;Brain CT results: No evidence of edema or bleeding. Calcifications of the bilateral carotid siphons, vertebral arteries and basal ganglia.;ASPECT score: 10;CT perfusion results: No evidence of hypoperfusion.;CTA results: No LVO evidence.;dyslipidemia: 0;hypertension: 0;diabetes mellitus: 1;chronic kidney disease: 0;smoking (0 = no, 1 = active, 2= past): 2;obesity/significant weight gain: 0;malignancy (past or present): 0;cerebrovascular attack: 0;heart failure: 0;arrhythmias: 0;family history of CAD: 0;thrombocytopenia: 298;peripheral vascular disease: 0;no significant medical history: IHD, COPD;LVD: 0;is there free >50% of the ICA: 1;is there a lesion in the MCA: 0;is there a lesion in the PCA: 0".
