# Supplementary material for: GPT-4 as a Clinical Decision Support Tool in Ischemic Stroke Management: Evaluation Study
Source: JMIR AI. 2025 Mar 7;4:e60391. doi: 10.2196/60391 (PMC11928773; doi:10.2196/60391)
Supplement: Multimedia Appendix 3 [file ai_v4i1e60391_app3.pdf]

Multimedia Appendix 3

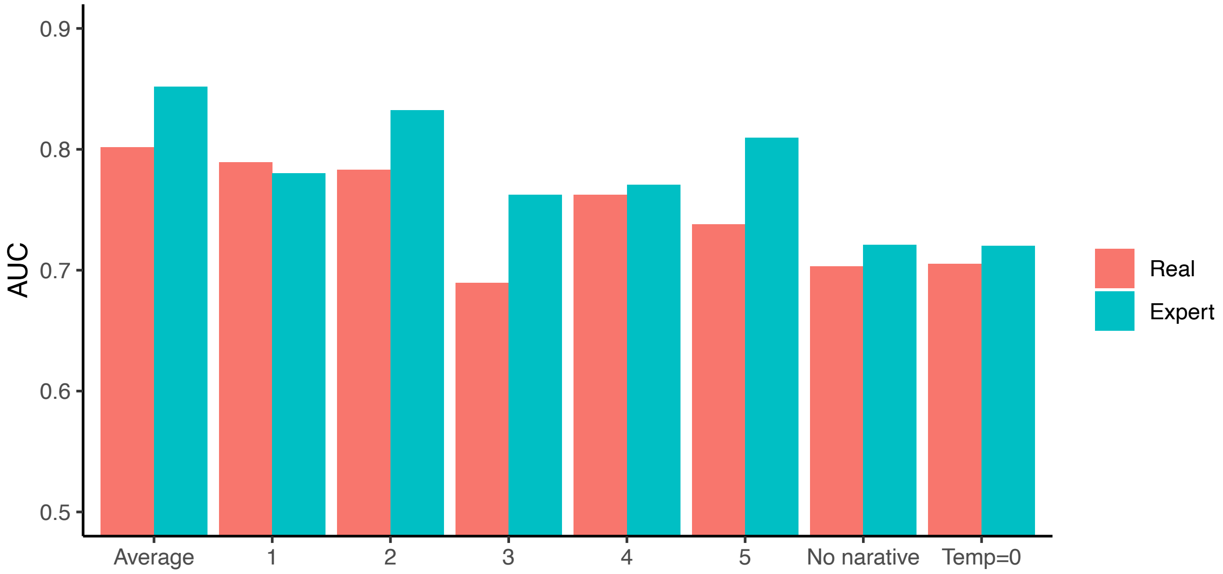

GPT-4 Assessments Performance. Area under the curve (AUC) for GPT-4 decision to treatment scores of each of the individual submissions (1-5) and the average. Each individual submission is lower than the average. In addition, we submitted the cases without the clinical presentation narrative, which yielded lower AUC (no narrative). Similarly, lower AUC was observed when cases were submitted with temperature=0.
